# *admixr*—R package for reproducible analyses using ADMIXTOOLS

**DOI:** 10.1093/bioinformatics/btz030

**Published:** 2019-01-22

**Authors:** Martin Petr, Benjamin Vernot, Janet Kelso

**Affiliations:** Department of Evolutionary Genetics, Max Planck Institute for Evolutionary Anthropology, Leipzig, Germany

## Abstract

**Summary:**

We present a new R package *admixr*, which provides a convenient interface for performing reproducible population genetic analyses (*f_3_*, *D*, *f_4_*, *f_4_*-ratio, *qpWave* and *qpAdm*), as implemented by command-line programs in the ADMIXTOOLS software suite. In a traditional ADMIXTOOLS workflow, the user must first generate a set of text configuration files tailored to each individual analysis, often using a combination of shell scripting and manual text editing. The non-tabular output files then need to be parsed to extract values of interest prior to further analyses. Our package simplifies this process by automating all low-level configuration and parsing steps, making analyses as simple as running a single R command. Furthermore, we provide a set of R functions for processing, filtering and manipulating datasets in the EIGENSTRAT format. By unifying all steps of the workflow under a single R framework, this package enables the automation of analytic pipelines, significantly improving the reproducibility of population genetic studies.

**Availability and implementation:**

The source code of the R package is available under the MIT license. Installation instructions, reference manual and a tutorial can be found on the package website at https://bioinf.eva.mpg.de/admixr.

**Supplementary information:**

Supplementary data are available at *Bioinformatics* online.

## 1 Introduction

The growing number of ancient and modern genome sequences have transformed our understanding of the evolutionary history of humans and other species. Several statistical methods have been developed to make inferences about past population movements and admixtures from genomic data. Chief among these has been a series of population genetic methods (*D*, $f_{3}$,$\;f_{4}$,$\;f_{4}$-ratio, *qpWave* and *qpAdm)* for estimating the amounts of genetic drift shared between populations, testing admixture hypotheses and estimating admixture proportions, implemented as command-line utilities in the ADMIXTOOLS software suite (Patterson *et al.*, 2012). Although ADMIXTOOLS has been used in many recent studies of human ancient DNA (Fu *et al.*, 2016; Haak *et al.*, 2015; Hajdinjak *et al.*, 2018; Lazaridis *et al.*, 2016), the tools in this package are rather cumbersome to use. First, each individual analysis or hypothesis test relies on a set of configuration files, which have to be generated using a combination of shell scripting and manual editing. Second, after running an ADMIXTOOLS command on the command-line, the user needs to extract relevant values from a non-tabular text file before they can be imported into software such as R for further analysis and plotting. This workflow is slow and potentially error-prone, especially if the user wishes to quickly iterate through different hypotheses involving many different populations or samples. Most importantly, however, it makes it challenging to conduct fully reproducible research. To overcome these challenges, we present a new R package for population admixture analyses which utilizes the ADMIXTOOLS software suite for the underlying calculations, but that provides a unified and convenient R interface. The package completely automates the generation, processing and parsing of all intermediate files, hiding all low-level details from the user, and allowing them to focus on the analysis itself. Importantly, unifying the entire analytic workflow in a single environment makes it possible to implement and share fully automated, reproducible analytic pipelines.

## 2 Implementation

The *admixr* package is implemented using the R programming language. It consists of several wrapper functions (calling ADMIXTOOLS commands internally from R), and a set of complementary functions for filtering and processing datasets in the EIGENSTRAT file format required by ADMIXTOOLS (Patterson *et al.*, 2012).

An EIGENSTRAT dataset is represented by an S3 object of the class EIGENSTRAT, which is created using the eigenstrat() constructor function, and encapsulates the paths to a trio of ‘ind’, ‘snp’ and ‘geno’ files:

> snps <- eigenstrat(“∼/path/to/eigenstrat/data”)

> snps

EIGENSTRAT object

=================

components:

 ind file: ∼/path/to/eigenstrat/data.ind

 snp file: ∼/path/to/eigenstrat/data.snp

 geno file: ∼/path/to/eigenstrat/data.geno

All other functions in the package accept this object as their first argument, and perform either a requested calculation on it (returning an R data frame for further analysis), or return a new, modified EIGENSTRAT S3 object (in case of filtering and processing functions) which can be used in additional downstream steps or calculations.

The core functionality of the package consists of the following set of R functions: f3(), d(), f4(), f4ratio(), qpWave() and qpAdm(), each implemented as a wrapper around one of the command-line programs distributed as part of the ADMIXTOOLS package.

## 3 Example usage

Performing even the most trivial analysis using ADMIXTOOLS presents a significant amount of overhead for the user. For example, to estimate the proportion of Neandertal ancestry in a set of individuals, X, the user would typically calculate an $f_{4}$-ratio statistic such as:

(1) $$\frac{f_{4}\left(Altai,\;Chimp;\;X,\;Mbuti\right)}{f_{4}\left(Altai,\;Chimp;\;Vindija,\;Mbuti\right)}.$$

The user first needs to create a file with a list of samples in each position of both *f_4_* statistics, a parameter file specifying the paths to a trio of EIGENSTRAT component files, then manually run the qpF4ratio command-line program, and then capture and parse its output to obtain relevant values (see Supplementary Information for a complete example workflow using a traditional ADMIXTOOLS approach). Note that changing the analysis setup [such as including a different set of populations in Equation (1)], performing the analysis on a subset of the genome, or modifying the analysis in another way, requires changes to be made to its configuration files. This presents a significant overhead for the user, especially when iterating through a complex set of population genetic hypotheses.

In contrast, using the *admixr* package, the same analysis can be performed with just the following snippet of R code:

 result <- f4ratio(

 X = c(“French”, “Han”, “Papuan”),

 A = “Altai”, B = “Vindija”, C = “Mbuti”, O =  “Chimp”,

 data = eigenstrat(“<path to EIGENSTRAT data>”)

 )

Internally, the f4ratio() function performs all configuration and parsing work, and returns an R data frame which can be immediately used for further statistical analysis and plotting:

> result

A B X C O alpha stderr Zscore

Altai Vindija French Mbuti Chimp 0.019696 0.003114  6.324

Altai Vindija Han Mbuti Chimp 0.024379 0.003364 7.248

Altai Vindija Papuan Mbuti Chimp 0.032167 0.003499 9.193

All other *admixr* wrapper functions have a similar interface and are described in the tutorial vignette on the package website in more detail.

## 4 Additional functionality

The fact that ADMIXTOOLS requires the data to be in EIGENSTRAT format presents additional challenges for quality control, processing and filtering, as this format is not supported by standard bioinformatics tools. Our R package therefore provides additional functionality to simplify the processing and filtering of EIGENSTRAT genotype data. This includes:

- Reading and writing of ind, snp and geno file components.
- Filtering of SNPs based on regions specified in a BED file.
- Restricting analyses to sites carrying transversion SNPs.
- Renaming samples or grouping them into larger population groups.
- Merging of EIGENSTRAT datasets.
- Counting the number of sites present or missing in each sample.

## Supplementary Material

btz030_Supplementary_Information
