# Supplementary material for: admixr—R package for reproducible analyses using ADMIXTOOLS
Source: Bioinformatics. 2019 Jan 22;35(17):3194–5. doi: 10.1093/bioinformatics/btz030 (PMC6736366; doi:10.1093/bioinformatics/btz030)
Supplement: btz030_Supplementary_Information [file btz030_supplementary_information.docx]

Supplementary Information

All files used in this document and its source in R Markdown can be found at: <https://github.com/bodkan/admixr-SI>.

A complete description of the functionality of the *admixr* package can be found in the tutorial vignette at: <https://bodkan.net/admixr/articles/tutorial.html>. In this supplement we provide an example analysis to demonstrate more clearly the distinctions between the traditional ADMIXTOOLS workflow and the *admixr* approach.

# Example analysis: Estimating the proportions of Neandertal ancestry

Our goal is to estimate the proportions of Neandertal ancestry in a set of present-day European individuals (French, Sardinian and Czech) using the following $f_{4}$-ratio statistic:

$$\alpha=\frac{f_{4}(Altai Neandertal, Chimpanzee; European X, African)}{f_{4}(Altai Neandertal, Chimpanzee; Vindija Neandertal, African)}.$$

## a) A traditional ADMIXTOOLS workflow

To perform this task using a traditional ADMIXTOOLS workflow, we have to do the following:

1. Create a pop file (f4ratio.pop).

This file specifies the order of populations in the $f_{4}$ statistic calculation above, and will contain the following (one $f_{4}$-ratio setup per row, as required by ADMIXTOOLS):

Altai Chimp : French Yoruba :: Altai Chimp : Vindija Yoruba
Altai Chimp : Sardinian Yoruba :: Altai Chimp : Vindija Yoruba
Altai Chimp : Czech Yoruba :: Altai Chimp : Vindija Yoruba

1. Create a par file (f4ratio_all.par).

This is the main configuration file and contains the paths to a trio of EIGENSTRAT components and a path to the pop file created in the previous step.

genotypename: ./data/snps.geno
snpname: ./data/snps.snp
indivname: ./data/snps.ind
popfilename: ./f4ratio.pop

1. Run the qpF4ratio command-line program and capture its output:

qpF4ratio -p f4ratio_all.par > f4ratio_all.log

1. Inspect the output file (f4ratio_all.log) and extract the relevant values.

To extract the values of interest (on lines beginning with “result:”), we could either copy-paste them manually into Excel, or extract them using a combination of grep/awk/sed and save them into a text file for further analysis (we will omit these steps here).

qpF4ratio: parameter file: f4ratio_all.par
### THE INPUT PARAMETERS
##PARAMETER NAME: VALUE
genotypename: ./data/snps.geno
snpname: ./data/snps.snp
indivname: ./data/snps.ind
popfilename: ./f4ratio.pop
## qpF4ratio version: 310
nplist: 3
 0 Altai 1
 1 Chimp 1
 2 French 1
 3 Yoruba 1
 4 Vindija 1
 5 Sardinian 1
 6 Czech 1
jackknife block size: 0.050
snps: 2055980 indivs: 7
number of blocks for block jackknife: 557
 alpha std. err Z (null=0)
 result: Altai Chimp French Yoruba : Altai Chimp Vindija Yoruba 0.021646 0.003738 5.791 9d
 result: Altai Chimp Sardinian Yoruba : Altai Chimp Vindija Yoruba 0.025142 0.003594 6.996 9d
 result: Altai Chimp Czech Yoruba : Altai Chimp Vindija Yoruba 0.026637 0.003605 7.389 9d
## end of run

## b) A new workflow using the *admixr* package

Running the following R code will perform all the low-level configuration and parsing work (steps 1-4 above) automatically, returning a simple R data frame:

> library(admixr)
>
> result <- f4ratio(
> X = c("French", "Sardinian", "Czech"),
> A = "Altai", B = "Vindija", C = "Yoruba", O = "Chimp",
> data = eigenstrat("data/snps")
> )

> result

# A tibble: 3 x 8
 A B X C O alpha stderr Zscore
 <chr> <chr> <chr> <chr> <chr> <dbl> <dbl> <dbl>
1 Altai Vindija French Yoruba Chimp 0.0216 0.00374 5.79
2 Altai Vindija Sardinian Yoruba Chimp 0.0251 0.00359 7.00
3 Altai Vindija Czech Yoruba Chimp 0.0266 0.00360 7.39

# Estimate Neandertal ancestry proportions on a subset of the data.

Let’s investiate a variation of the analysis above, this time estimating Neandertal ancestry proportions in a subset of the genome (in a set of regions specified in a BED file regions.bed).

## a) A traditional ADMIXTOOLS workflow

All ADMIXTOOLS commands accept a parameter badsnpname, which can be specified in a par file and instructs ADMIXTOOLS to ignore SNPs at specified coordinates.

In a traditional ADMIXTOOLS workflow, we would have to create this file using some form of shell scripting. For example, we could do the following:

1. Convert the EIGENSTRAT snp file into a BED format:

awk -v OFS='\t' '{print $2, $4 - 1, $4, $0}' data/snps.snp > snps.bed

1. Intersect the newly generated BED file with coordinates of regions in reigons.bed. We want to keep only those sites that fall outside of those regions and saving them in a snp format again.

bedtools intersect -v -a snps.bed -b regions.bed \
 | cut -f 4- \
 > excluded_sites.snp

1. Create a new par file (f4ratio_subset.par), with a new parameter badsnpname:

genotypename: ./data/snps.geno
snpname: ./data/snps.snp
indivname: ./data/snps.ind
popfilename: ./f4ratio.pop
badsnpname: ./excluded_sites.snp

1. Run qpF4ratio with the new par file and capture its output:

qpF4ratio -p f4ratio_subset.par > f4ratio_subset.log

1. Extract the relevant values from the output file (f4ratio_subset.log):

qpF4ratio: parameter file: f4ratio_subset.par
### THE INPUT PARAMETERS
##PARAMETER NAME: VALUE
genotypename: ./data/snps.geno
snpname: ./data/snps.snp
indivname: ./data/snps.ind
popfilename: ./f4ratio.pop
badsnpname: ./excluded_sites.snp
## qpF4ratio version: 310
nplist: 3
 0 Altai 1
 1 Chimp 1
 2 French 1
 3 Yoruba 1
 4 Vindija 1
 5 Sardinian 1
 6 Czech 1
jackknife block size: 0.050
snps: 1716304 indivs: 7
number of blocks for block jackknife: 556
 alpha std. err Z (null=0)
 result: Altai Chimp French Yoruba : Altai Chimp Vindija Yoruba 0.023227 0.003882 5.984 9d
 result: Altai Chimp Sardinian Yoruba : Altai Chimp Vindija Yoruba 0.027174 0.003799 7.154 9d
 result: Altai Chimp Czech Yoruba : Altai Chimp Vindija Yoruba 0.027380 0.003821 7.165 9d
## end of run

## b) A new workflow using the *admixr* package

Using the *admixr* package, we can use the function filter_bed() to do all filtering automatically:

> library(admixr)
>
> snps <- eigenstrat("data/snps")
> subset <- filter_bed(snps, "regions.bed") # create a new EIGENSTRAT object
>
> result <- f4ratio(
> X = c("French", "Sardinian", "Czech"),
> A = "Altai", B = "Vindija", C = "Yoruba", O = "Chimp",
> data = subset
> )

> result

# A tibble: 3 x 8
 A B X C O alpha stderr Zscore
 <chr> <chr> <chr> <chr> <chr> <dbl> <dbl> <dbl>
1 Altai Vindija French Yoruba Chimp 0.0232 0.00388 5.98
2 Altai Vindija Sardinian Yoruba Chimp 0.0272 0.00380 7.15
3 Altai Vindija Czech Yoruba Chimp 0.0274 0.00382 7.16

Furthermore, because all *admixr* functions are inspired by the “tidyverse philosophy”, we can utilize the %>% pipe operator from the magrittr package and run the whole analysis in one step:

> library(admixr)
> library(magrittr)
>
> result <-
> eigenstrat("data/snps") %>%
> filter_bed("regions.bed") %>%
> f4ratio(
> X = c("French", "Sardinian", "Czech"),
> A = "Altai", B = "Vindija", C = "Yoruba", O = "Chimp"
> )
>
> result

# A tibble: 3 x 8
 A B X C O alpha stderr Zscore
 <chr> <chr> <chr> <chr> <chr> <dbl> <dbl> <dbl>
1 Altai Vindija French Yoruba Chimp 0.0232 0.00388 5.98
2 Altai Vindija Sardinian Yoruba Chimp 0.0272 0.00380 7.15
3 Altai Vindija Czech Yoruba Chimp 0.0274 0.00382 7.16

# A more complex example

The “pipe-friendly” nature of *admixr* makes it possible to compose even more complex pipelines, such as the following:

> library(admixr)
> library(magrittr)
>
> result <-
> eigenstrat("data/snps") %>%
> relabel(Europeans = c("French", "Sardinian", "Czech")) %>%
> filter_bed("regions.bed") %>%
> transversions_only() %>%
> f4ratio(
> X = "Europeans",
> A = "Altai", B = "Vindija", C = "Yoruba", O = "Chimp"
> )

> result

# A tibble: 1 x 8
 A B X C O alpha stderr Zscore
 <chr> <chr> <chr> <chr> <chr> <dbl> <dbl> <dbl>
1 Altai Vindija Europeans Yoruba Chimp 0.0282 0.00411 6.85

Accomplishing the same task using shell scripting would require a significant amount of work.
